# Integrated docking–molecular dynamics, ADME–toxicity profiling, and transcriptomic validation identify oroxylin a as a TLR7-targeting flavonoid candidate in systemic lupus erythematosus

**DOI:** 10.3389/fchem.2026.1863132

**Published:** 2026-07-01

**Authors:** Nourah Albarrak, Hanadi Albjeedi, Ebtihal Kamal, Mohammed F. Aldawsari, Hisham N. Altayb, Ehssan Moglad

**Affiliations:** 1 Department of Pharmaceutics, College of Pharmacy, Prince Sattam Bin Abdulaziz University, Al-Kharj, Saudi Arabia; 2 Department of Basic Medical Sciences, College of Medicine, Prince Sattam Bin Abdulaziz University, Al Kharj, Saudi Arabia; 3 Department of Biochemistry, Faculty of Science, King Abdulaziz University, Jeddah, Saudi Arabia

**Keywords:** Apigenin, Baicalein, Bavachinin, Oroxylin a, SLE, Wogonin

## Abstract

**Background:**

Systemic lupus erythematosus (SLE) remains difficult to control despite current therapies, highlighting the need for more selective, mechanism-based treatments targeting key innate immune components such as Toll-like receptor 7 (TLR7).

**Objectives:**

To identify natural flavonoid compounds with favorable stability and drug-like properties as hypothesis-generating TLR7 inhibitors for SLE using an integrated *in silico* workflow.

**Methods:**

Six flavonoids (bavachinin, baicalein, apigenin, wogonin, eupalitin, and oroxylin A) were screened against TLR7 using molecular docking and 100-ns molecular dynamics simulations of the two top-scoring complexes. Drug likeness and ADME-toxicity were assessed using *in silico* toxicity models. KEGG pathway enrichment of predicted flavonoid targets was performed, and two independent SLE GEO datasets (GSE65391 and GSE61635) were analyzed to define upregulated genes and identify overlap with predicted targets retrieved from SwissTargetPrediction.

**Results:**

Docking identified bavachinin and oroxylin A as the strongest initial binders to TLR7, while molecular dynamics showed that the oroxylin A–TLR7 complex exhibited greater conformational stability and more persistent interactions than bavachinin. Both ligands met drug-likeness criteria, but oroxylin A displayed a more balanced ADME–toxicity profile and less toxicity than the more lipophilic, immunotoxic bavachinin. KEGG enrichment of predicted oroxylin A targets highlighted inflammatory and immune-related pathways, and 12 predicted oroxylin A targets were consistently upregulated across both SLE datasets.

**Conclusion:**

Oroxylin A showed the most favorable combination of TLR7 complex stability, ADME–toxicity properties, immune-related pathway enrichment, and overlap with SLE-upregulated genes, supporting its prioritization as a potential TLR7-directed, hypothesis-generating candidate for SLE that warrants rigorous *in vitro* and *in vivo* validation.

## 1 Introduction

Systemic Lupus Erythematosus (SLE) is a complex, chronic autoimmune disease characterized by the immune system attacking the body’s own tissues, leading to widespread inflammation and damage in multiple organs (Ameer et al., 2022). Hydroxychloroquine (HCQ) remains a cornerstone therapy owing to its immunomodulatory properties; however, many patients continue to experience disease flares even with good adherence. This highlights the need for more selective and effective therapeutic strategies (Jorge et al., 2022).

Toll-like receptor 7 (TLR7) has emerged as a promising molecular target in SLE. TLR7 recognizes RNA-containing immune complexes and drives downstream inflammatory signaling. Its activation promotes type I interferon and autoantibody production, central features of SLE pathogenesis. Furthermore, TLR7 activation, together with B-cell–activating factor (BAFF) and interferon-γ (IFN-γ), supports the survival and expansion of autoreactive B-cells, thereby contributing to lupus flares (Satterthwaite, 2021; von Hofsten et al., 2024). In parallel, gain-of-function variants and overactivation of TLR7 have been increasingly linked to lupus-like autoimmunity in humans and mice, reinforcing the rationale for carefully designed TLR7-directed interventions (Brown et al., 2022; David et al., 2024; Parvaneh et al., 2026; Sethumadhavan et al., 2026; Zeng et al., 2025). These findings highlight TLR7 as a potential immunological node and a novel target for therapeutic intervention. A range of TLR7 antagonists, including synthetic oligonucleotides and small molecules, are under investigation to inhibit the endosomal TLR7 signaling (Kandimalla et al., 2013; Tojo et al., 2020). However, these approaches are frequently limited by incomplete selectivity for TLR7 over other endosomal TLRs (Haseeb et al., 2024; Padilla-Salinas et al., 2019).

Natural flavonoids represent a valuable source of bioactive compounds due to their structural diversity and established pharmacological activities. Over recent decades, they have contributed substantially to drug discovery in oncology, infectious diseases, and immunology (Newman and Cragg, 2020). Despite the long-standing interest in natural products as immunomodulatory scaffolds, natural flavonoids have not, to our knowledge, been systematically evaluated against TLR7 in the context of SLE using integrated molecular docking, molecular dynamics, ADME/toxicity profiling, chemotype analysis, and pathway-level target enrichment. To explore TLR7-directed natural inhibitors in, we selected a small panel of six structurally related flavonoids (bavachinin, baicalein, wogonin, eupalitin, apigenin, and oroxylin A) with known anti-inflammatory and immunomodulatory activities, rather than performing a large-scale virtual screen.

Bavachinin, derived from Psoralea corylifolia, exhibits antiviral, anticancer, antibiotic, and anti-inflammatory effects (Atlasbaf et al., 2025). Baicalein from Scutellaria baicalensis shows antioxidant and anti-inflammatory properties relevant to immune dysregulation (Seo et al., 2011; Zen et al., 2022). Moreover, research indicates that wogonin has demonstrated immunomodulatory activity through regulation of cytokine signaling (Błach-Olszewska et al., 2008). Eupalitin, a methylated flavonoid from Boerhaavia diffusa, exhibits antioxidant activity that may ameliorate immune-mediated damage (Baig et al., 2018). Apigenin, found in chamomile and parsley, modulates inflammatory cytokine production (Jabir et al., 2021). Oroxylin A, isolated from Scutellaria baicalensis, has been reported to exert anti-inflammatory and cytoprotective effects (Sajeev et al., 2022).

In many previous *in silico* investigations of natural products, including flavonoids, screening efforts have centered on small sets of synthetic agonists or antagonists and largely relied on single-step molecular docking or short simulations of individual compounds, without systematic integration of long-timescale molecular dynamics, ADME-toxicity profiling, and target-level pathway analysis (Abiri et al., 2021; El Khatabi et al., 2022; Gentile et al., 2015; Khattabi et al., 2024). Such approaches can identify ligands with favorable docking scores but are often insufficient to assess the conformational stability of the complex, the pharmacokinetics and safety of the candidate molecules, or their function in immune and cytokine signaling associated with SLE. To address these gap, we aimed to prioritize flavonoids with documented anti-inflammatory effects and established roles in the immune system that are suitable for downstream mechanistic evaluation within a hypothesis-generating computational workflow. In this study, we used integrated structure-based molecular docking, 100-ns molecular dynamics simulations (MDS), drug-likeness prediction, *in silico* ADME/toxicity evaluation, cheminformatics-based chemical similarity mapping, and KEGG pathway enrichment of predicted targets to enable rapid screening of the six structurally related, anti-inflammatory flavonoids (bavachinin, baicalein, apigenin, wogonin, eupalitin, and oroxylin A). This multistep approach predicts the top-ranking flavonoid as a TLR7-directed hypothesis-generating compound and compares its stability against closely related flavonoids that display divergent ADME–toxicity and pathway signatures.

To the best of our knowledge, this is the first study to systematically evaluate a panel of structurally related, anti-inflammatory flavonoids for their TLR7 binding potential in SLE using an integrated workflow of molecular docking, molecular dynamics, and ADME–toxicity prediction.

## 2 Methodology

**FIGURE 1:**
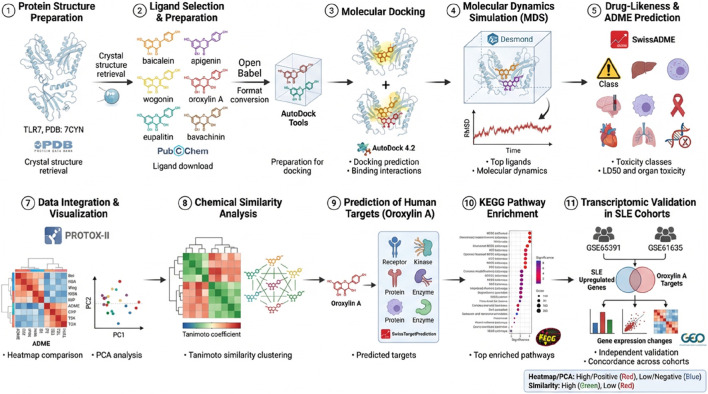
Workflow of the study.

All the steps of this study were summarized in Figure 1.

### 2.1 Preparation of protein structure

The cryo-electron microscopy (cryo-EM) of TLR7 (TLR7; PDB ID: 7CYN, uniprot ID: Q9NYK1) was retrieved from the Protein Data Bank (Morris et al., 2009). Only the ectodomain chain of TLR7 (chain A) was retained for docking, grid generation and subsequent solvation during molecular dynamics simulations. Protein preparation was performed using AutoDock Tools 1.5.7 (ADT). Co-crystallized ligands and non-essential heteroatoms, including crystallographic water molecules, were removed. Polar hydrogen atoms were added, and Kollman charges were assigned. The prepared receptor structure was saved in PDBQT format for molecular docking.

### 2.2 Ligand selection and preparation

The structures of the selected flavonoids, baicalein, apigenin, wogonin, oroxylin A, eupalitin, and bavachinin, were downloaded in SDF format from PubChem (https://pubchem.ncbi.nlm.nih.gov/) (Supplementary Table S1) (Kim et al., 2025). The SDF files were converted to PDB format using Open Babel, which was also used to remove salt fragments, add missing hydrogens, and perform basic structural cleanup (O’Boyle et al., 2011).

Ligands were then imported into AutoDock Tools, where non-polar hydrogens were merged, Gasteiger atomic charges were assigned, and rotatable bonds were defined. Each ligand was saved in PDBQT format for docking.

### 2.3 Molecular docking study

Molecular docking studies were conducted using AutoDock 4.2 to predict the probable binding interactions between TLR7 and the selected ligands (Morris et al., 2009).

#### 2.3.1 Grid parameter generation

AutoGrid (AutoDock 4.2) was used to generate grid maps covering the TLR7 binding site. The grid box was centered on the active-site residues, with the grid center coordinates set to 15.386, −13.842, and 12.964 Å along the X, Y, and Z-axes, respectively, and dimensions chosen to fully enclose the ligand-binding pocket. A grid spacing of 0.375 Å was applied, which corresponds to the default resolution and provides a balance between computational efficiency and accurate sampling of protein–ligand interactions. During docking, the TLR7 receptor was treated as rigid, and only ligand torsions were allowed to rotate, consistent with the standard Lamarckian Genetic Algorithm (LGA) protocol which is implemented in AutoDock.

#### 2.3.2 Docking procedure

Docking simulations were performed using the LGA, the standard search algorithm in AutoDock 4.2. Receptor and ligand PDBQT files were loaded into AutoDock, and docking simulations were run using default parameters unless otherwise stated. Docking log files (DLGs) were generated.

#### 2.3.3 Analysis of docking results

DLGs were analyzed to obtain binding affinity values ΔG (kcal/mol). Docked poses were clustered using an RMSD tolerance of 2.0 Å. For each ligand, the pose with the lowest binding energy was selected as the most representative binding conformation.

### 2.4 Molecular dynamics simulation

The two top-ranked ligands from docking were subjected to molecular dynamics simulations (MDS) using the Desmond module based on docking performance and to prioritize the most promising candidates for more intensive stability analysis. Each TLR7–ligand complex was solvated in an orthorhombic water box extending 10 Å in all directions, using the TIP4P water model. Counterions were added to neutralize the system.

Energy minimization was carried out using the OPLS3e force field for 2000 iterations. Production MD simulations were performed for 100 ns under NPT ensemble conditions at 300 K and 1.013 bar, using the Nosé–Hoover thermostat with a relaxation time of 1 ps and a 2-fs time step (Bowers et al., 2006). Snapshots were recorded every 100 ps? Trajectories were analyzed using the Maestro Simulation Interaction Diagram tool.

### 2.5 Prediction of drug-likeness and ADME properties

SwissADME (http://www.swissadme.ch) (Daina et al., 2017) was used to predict drug-likeness and pharmacokinetic properties (Daina et al., 2017). Lipinski’s Rule of Five was applied to evaluate molecular weight, hydrogen bond donors and acceptors, and lipophilicity (log P). Compounds that violate two or more rules were classified as having poor drug-likeness.

Radar plots were generated to visualize physicochemical properties associated with oral bioavailability. ADME parameters including lipophilicity, aqueous solubility, gastrointestinal absorption, blood–brain barrier penetration, skin permeation, and cytochrome P450 enzyme inhibition were also obtained.

### 2.6 Toxicity prediction

The toxicity of the top-ranking compounds was predicted using the PROTOX-II web server (https://tox-new.charite.de) (Banerjee et al., 2024). Predicted parameters included toxicity class, the median lethal dose (LD50), hepatotoxicity, cytotoxicity, immunotoxicity, carcinogenicity, cardiotoxicity, respiratory toxicity, and mutagenicity (Banerjee et al., 2018).

### 2.7 ADME–toxicity data integration and visualization

Drug-likeness, physicochemical, and ADME parameters along with the *in silico* toxicity indices were used to generate a matrix with ligands as rows and ADME–tox features as columns, then scaled to zero mean and unit variance in R (version 4.3.2) to allow comparison across different numeric ranges. A heatmap was generated using the pheatmap package to visually compare ADME–toxicity profiles across the flavonoid panel.

### 2.8 Principal component analysis of ADME–toxicity properties

A set of numeric physicochemical, ADME, and toxicity-related descriptors of each compound was retrieved from SwissADME and ProTox-II and transformed into a matrix with only numeric descriptors. Mean centering and scaling to unit variance were performed on each descriptor using R software (version 4.3.2). Principal component analysis (PCA) was carried out on the dataset with the base R function prcomp. Scores of the first two principal components were plotted with the ggplot2 package, with each ligand given a different color to represent its location with regard to other ligands in ADME-toxicity property space.

### 2.9 Chemical similarity analysis

Chemical similarity among the six flavonoids was evaluated by retrieving the three-dimensional structures of baicalein, apigenin, wogonin, oroxylin A, eupalitin, and bavachinin and downloading them in SDF format from PubChem and importing them into R (version 4.3.2) using the ChemmineR Bioconductor package. Atom-pair fingerprints were generated for each ligand with sdf2ap, and pairwise Tanimoto coefficients were calculated to obtain a symmetric chemical similarity matrix.

Hierarchical clustering of the resulting distance matrix (1 − Tanimoto coefficient) was performed using the average-linkage method implemented in the base hclust function in R. The dendrogram was generated to identify clusters of structurally related flavonoids and to determine the relative positioning of the predicted two top-ranking compounds within the chemical space of the panel. A heatmap of the clustering results is provided to show the Tanimoto similarity coefficients between the six flavonoids.

### 2.10 Prediction of human targets of the predicted top-ranking flavonoid compound

The human protein targets of the predicted top-ranking flavonoid were retrieved from SwissTargetPrediction (https://www.swisstargetprediction.ch/) (Daina et al., 2019). SwissTargetPrediction infers likely targets based on 2D/3D chemical similarity to known ligands; as such, the resulting target list represents a probabilistic, similarity-based prioritization. We submitted the SDF file of the predicted top-ranking flavonoid, specifying *Homo sapiens* as the target organism.

### 2.11 KEGG pathway enrichment analysis of the targets of the predicted top-ranking flavonoid

Functional enrichment analysis of the predicted top-ranking flavonoid targets was performed in R using the clusterProfiler package in R (version 4.3.2). Gene symbols were mapped to Entrez Gene identifiers using org. Hs.e.g.,.db (bitr function, fromType = “SYMBOL,” toType = “ENTREZID”). KEGG overrepresentation analysis was carried out with enrichKEGG (organism = “hsa,” keyType = “ncbi-geneid,” pvalueCutoff = 0.05, qvalueCutoff = 0.2), using all human Entrez genes from org. Hs.e.g.,.db as the background. Enriched pathways were ranked by adjusted p value (Benjamini–Hochberg), and gene–pathway relationships were summarized. Dot plots of the top enriched KEGG pathways were generated with clusterProfiler’s dotplot function and ggplot2 for visualization.

### 2.12 Independent transcriptomic validation of predicted oroxylin a targets in public SLE cohorts

To provide a validation layer for the *in silico* findings, an independent transcriptomic analysis was performed using two whole-blood SLE cohorts (GSE65391, Illumina HumanHT-12 V^4^.0, GSE65391; and GSE61635, Affymetrix Human Genome U133 Plus 2.0, GPL570) from the Gene Expression Omnibus (GEO). Normalized series matrix files and associated phenotype data were downloaded using the GEOquery package in R (getGEO, GSEMatrix = TRUE), and the processed expression values provided by the original studies were used directly. Expression values were checked for log2 scaling. Probe identifiers were mapped to official gene symbols using the platform-specific annotation files; when multiple probes mapped to the same gene, the probe with the highest median expression across all samples was retained to obtain a single expression value per gene. Samples were classified as SLE cases or healthy controls using the GEO phenotype annotations, and for each dataset, we summarized group-wise expression differences as log2(SLE/control) contrasts for genes of interest rather than performing a full genome-wide differential expression analysis. Log2 fold-change estimates for oroxylin A target genes and selected pathway-level readouts were extracted separately from GSE65391 and GSE61635 and visualized using bar plots, scatterplots, and heatmaps generated with ggplot2 and pheatmap to assess the direction and relative magnitude of expression changes across cohorts. This transcriptomic comparison was used as an exploratory, supportive validation step linking the *in silico* predictions to the disease-relevant whole-blood signatures in SLE.

## 3 Results

### 3.1 Molecular docking analysis

**FIGURE 2:**
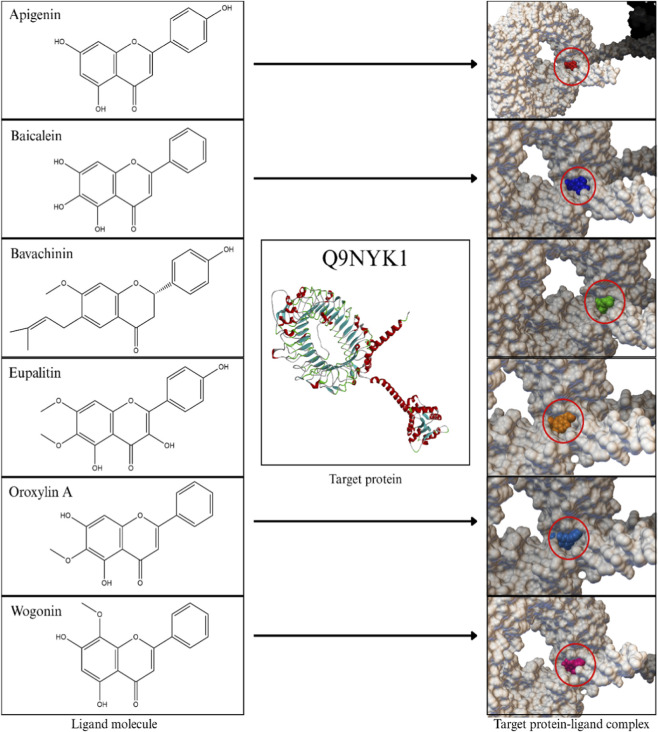
Simulated docking sites of TLR7.

Molecular docking was carried out to evaluate the binding affinities of the six flavonoid compounds towards the active site of TLR7; the predicted binding orientations are shown in Figure 2.

**FIGURE 3:**
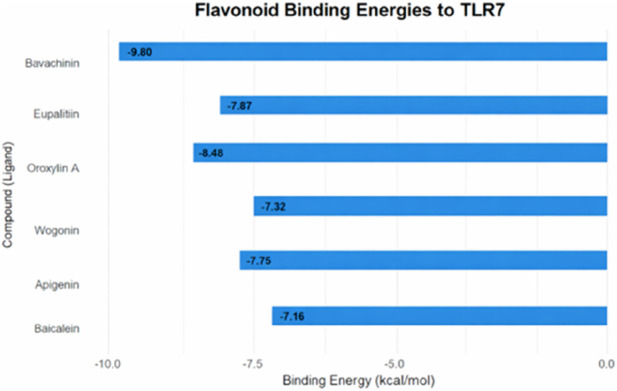
Binding energies of flavonoids to TLR7. Horizontal bar chart showing the predicted binding energies (ΔG, kcal/mol) of the six screened flavonoids docked into the predicted TLR7 binding site. Bavachinin exhibited the lowest docking energy (−9.80 kcal/mol), followed by oroxylin A (−8.48 kcal/mol) and eupalitin (−7.87 kcal/mol), whereas baicalein, apigenin, and wogonin showed comparatively weaker binding (−7.16 to −7.75 kcal/mol). These values indicate that bavachinin and oroxylin A form the most energetically favorable predicted complexes with TLR7 among the tested compounds.

Bavachinin demonstrated the strongest binding affinity to TLR7 (−9.80 kcal/mol), followed by oroxylin A (−8.48 kcal/mol), while baicalein exhibited the weakest affinity (−7.16 kcal/mol) (Figure 3). These docking results, therefore, initially favored bavachinin as the top-ranked TLR7 binder, with oroxylin A as the second-ranked ligand, therefore selected for molecular dynamics simulations.

### 3.2 Hydrogen bond interaction analysis

Hydrogen-bond interactions were analyzed to understand the structural basis for the observed docking affinities. Bavachinin formed three hydrogen bonds within the predicted TLR7 binding site, consistent with its highest predicted binding affinity. Oroxylin A formed 3 hydrogen bonds, while eupalitin formed two hydrogen bonds. Other ligands with lower predicted binding affinity generally formed fewer hydrogen bonds, supporting the role of hydrogen bonding in ligand stabilization (Table 1).

**TABLE 1:** Hydrogen-bond interactions formed between flavonoids and key residues within the predicted TLR7 binding site as predicted by molecular docking.

Compound	TLR7 H-bonds	TLR7 residues
Baicalein	1	ILE805
Apigenin	2	LYS54, ILE805
Wogonin	2	LEU34, TYR807
Oroxylin A	3	TYR807, ASP37, LEU34
Eupalitin	2	THR810, LYS54
Bavachinin	3	LYS54, ILE805 THR810

Table 1 summarizes hydrogen-bond interactions identified between the investigated ligands and TLR7. Other interaction types, including hydrophobic, and water-mediated interactions, are discussed separately in the two-dimensional interaction analysis and are therefore not included in this table.

### 3.3 Result of molecular dynamics simulations

**FIGURE 4:**
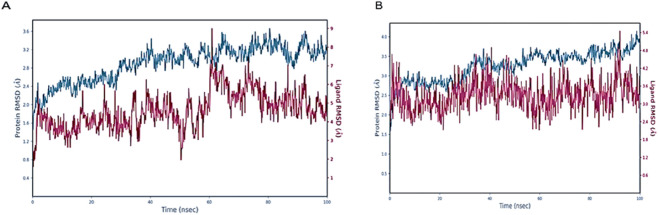
RMSD generated from molecular dynamics simulation analysis of ligand–TLR7 interactions over a 100 ns trajectory. *The ligand is shown in red, and the protein backbone is depicted in blue. Interactions of TLR7 and Bavachinin* **(A)** *and oroxylin A* **(B)** *.*

Molecular dynamics simulations were performed for the bavachinin–TLR7 and oroxylin A–TLR7 complexes to assess whether the top docking scores translated into stable binding over time. For bavachinin–TLR7, the protein backbone stabilized with RMSD values between 3.0 and 3.5 Å, but ligand RMSD increased markedly after approximately 15 ns, reaching up to ∼9 Å, suggesting partial disengagement from the binding site (Figure 4A **)**. In contrast, the oroxylin A–TLR7 complex showed greater stability, with protein backbone RMSD within 2.0–2.8 Å and moderate ligand RMSD (3–6 Å) (Figure 4B), indicating more consistent binding. Taken together, these simulations indicate that, although bavachinin is favored at the docking stage, oroxylin A forms the more dynamically stable TLR7 complex over the 100-ns trajectory, suggesting that MD stability rather than docking score alone should guide prioritization of flavonoid candidates.

**FIGURE 5:**
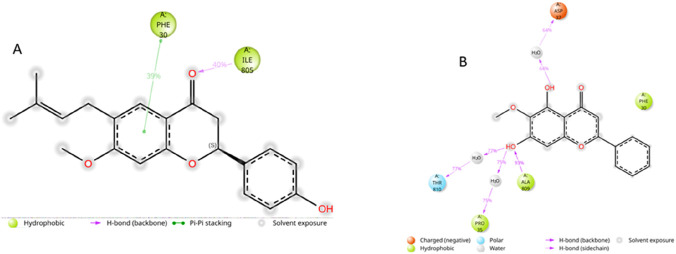
2D interaction illustrating the interactions between TLR7 residues and ligand atoms during molecular dynamics simulation over a 100 ns trajectory. Hydrogen bonds are shown in violet arrows and hydrophobic interactions in red. Residues are colored according to their charges. Interactions of TLR7 and Bavachinin **(A)** and oroxylin A **(B)**.

**FIGURE 6:**
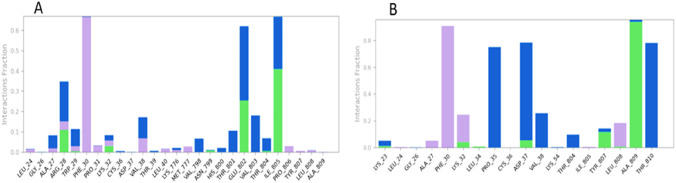
Interaction frequency histograms depicting hydrogen bonds, hydrophobic contacts, water bridges, and ionic interactions between TLR7 residues and **(A)** bavachinin and **(B)** oroxylin A over 100-ns molecular dynamics simulations.

Two-dimensional interaction profiles revealed that bavachinin formed predominantly hydrophobic contacts (notably with Phe30 and Ile805), whereas oroxylin A maintained persistent hydrogen bonds and polar interactions involving residues such as Asp37, Thr810, Pro35, Ala809, and several water-mediated contacts (Figures 5, 6).

A summary of docking energies, hydrogen-bond counts, and qualitative MD stability for the flavonoid panel is provided in Table 2. This comparison highlights that bavachinin, despite its lowest docking ΔG, displays reduced dynamic stability relative to Oroxylin A, whereas Oroxylin A combines favorable docking energy with a more persistent TLR7 interaction profile.

**TABLE 2:** Summary of docking and molecular dynamics parameters for TLR7–flavonoid complexes.

Compound	Docking binding energy (kcal/mol)	H-bonds in docking pose	MD stability (100 ns)	Key interacting residues (MD; top ligands)
Bavachinin	−9.80	3	Lower: Ligand RMSD ↑ to ∼9 Å, partial disengagement after ∼15 ns	Lys54, Ile805, Thr810 (docking); transient contacts with Ile805, Val803, Val798, Leu34, Val38 Ala809, Leu40 in early trajectory
Oroxylin A	−8.48	3	Higher: Backbone RMSD 2.0–2.8 Å; ligand RMSD 3–6 Å; persistent binding	Tyr807, Asp37, Leu34 (docking); stable hydrophobic contacts with Leu34, Val38, Val798, Val803, Ala809, Leu40; and H-bonds with Tyr807
Eupalitin	−7.87	2	Not simulated	Thr810, Lys54 (docking)
Baicalein	−7.16	1	Not simulated	Ile805 (docking)
Apigenin	−7.75	2	Not simulated	Lys54, Ile805 (docking)
Wogonin	−7.32	2	Not simulated	Leu34, Tyr807 (docking)

### 3.4 Result of ADME analysis

ADME and drug-likeness properties of oroxylin A were evaluated using SwissADME. They complied with Lipinski’s Rule of Five, indicating favorable oral drug likeness (Supplementary Table S2), and the radar plot illustrated physicochemical properties consistent with acceptable oral bioavailability (Supplementary Figure S1).

### 3.5 Results of in silico toxicity prediction and comparative risk analysis

Toxicity prediction using PROTOX-II showed that both bavachinin and oroxylin A demonstrated respiratory toxicity risk, with bavachinin additionally showing predicted immunotoxicity and oroxylin A showing predicted cardiotoxicity (Supplementary Table S3). Neither compound demonstrated predicted hepatotoxicity, cytotoxicity, or carcinogenicity. Radar chart of oroxylin A with the mean values of active compounds in the dataset (Supplementary Figure S1).

### 3.6 Comparative ADME–toxicity profiles of flavonoids

**FIGURE 7:**
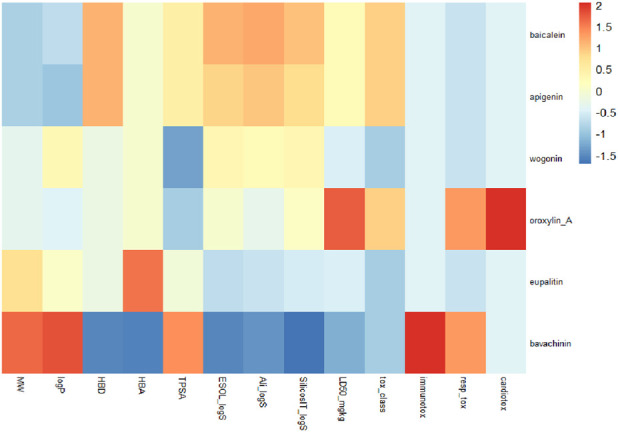
ADME–toxicity profiles of the screened flavonoids Heatmap showing scaled ADME and *in silico* toxicity parameters for the six flavonoids evaluated against TLR7. Rows correspond to individual ligands, and columns to physicochemical and toxicity-related descriptors, including molecular weight (MW), consensus logP, hydrogen bond donors (HBD) and acceptors (HBA), topological polar surface area (TPSA), three aqueous solubility estimates (ESOL_logS, Ali_logS, and SilicosIT_logS), predicted oral lethal dose (LD50_mgkg), overall toxicity class (tox_class), and binary *in silico* flags for immunotoxicity (immunotox), respiratory toxicity (resp_tox), and cardiotoxicity (cardiotox), derived from SwissADME and ProTox-II outputs. Colors represent z-score–scaled values for each descriptor across ligands, with red indicating relatively higher and blue relatively lower values, enabling visual comparison of ADME–toxicity patterns within the flavonoid panel.

The heatmap of scaled ADME–toxicity parameters revealed clear differences among the six flavonoids (Figure 7). Bavachinin showed relatively high molecular weight and consensus logP, together with relatively low topological polar surface area (TPSA) and a distinct solubility pattern, consistent with a more lipophilic profile. Oroxylin A displayed a comparatively lower molecular weight and moderate lipophilicity, with intermediate hydrogen-bond counts and TPSA, placing it in a central region of physicochemical space compared with the other ligands. Both bavachinin and oroxylin A were highlighted by the heatmap through positive respiratory toxicity signals, with bavachinin additionally marked for immunotoxicity and oroxylin A for cardiotoxicity, whereas the remaining flavonoids showed a comparatively benign *in silico* toxicity pattern.

### 3.7 Multivariate analysis of flavonoid ADME–toxicity profiles

**FIGURE 8:**
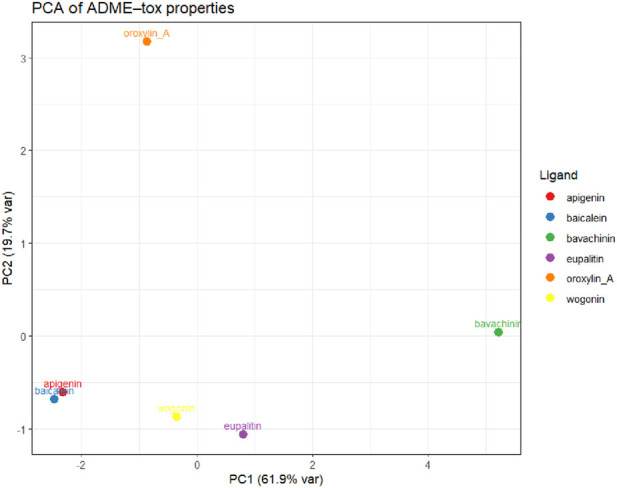
Principal component analysis of ADME–toxicity properties of the flavonoid panel Scatter plot of the first two principal components (PC1 and PC2) derived from standardized ADME–toxicity descriptors of the six flavonoids evaluated against TLR7. Each point represents one ligand (apigenin, baicalein, bavachinin, eupalitin, oroxylin A, or wogonin), colored according to the legend on the right. PC1 explains 61.9% and PC2 19.7% of the total variance, jointly capturing most of the variation in molecular weight, consensus logP, hydrogen bond donors and acceptors, topological polar surface area, solubility indices, LD50, toxicity class, and organ-specific toxicity flags obtained from SwissADME and ProTox-II. The separation of bavachinin along PC1 and of oroxylin A along PC2 illustrates their distinct multivariate ADME–toxicity profiles relative to the other flavonoids.

The PCA of standardized ADME–toxicity descriptors indicated that the first two principal components contributed to approximately 81.6% of the total variance (PC1: 61.9%, PC2: 19.7%), and bavachinin was projected strongly along PC1, indicating its significantly distinct ADME–toxicity profile in terms of its higher molecular weight, lipophilicity, and solubility/toxicity parameters compared with all other flavonoids. Oroxylin A was projected along PC2 and indicated its distinct ADME-toxicity profile in terms of its moderate molecular weight and logP values, as well as its TPSA and solubility indices and organ toxicity flags, compared with all other studied flavonoids. On the other hand, baicalein and apigenin were found to be close to each other at negative values of PC1, and wogonin and eupalitin were found at intermediate values of PC1 and PC2 (Figure 8).

The separation of bavachinin along PC1 and oroxylin A along PC2, therefore, reflects their distinct physicochemical properties, with the hydrophobic bavachinin more driving its extreme profile and oroxylin A’s 5,7-dihydroxy-6-methoxy flavone core underpinning its intermediate molecular size, logP, TPSA, and organ-toxicity indices.

Thus, the PCA separates compounds primarily according to their multivariate ADME–toxicity profiles, highlighting bavachinin as an ADME–toxicity outlier and oroxylin A as occupying a distinct, intermediate region, while the remaining flavonoids show more similar pharmacokinetic/toxicity properties.

### 3.8 Chemical similarity of flavonoids

**FIGURE 9:**
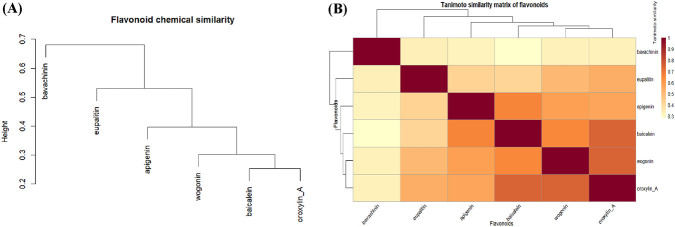
Flavonoid chemical similarity. **(A)** Dendrogram, **(B)** Clustered Tanimoto similarity heatmap **(A)** Hierarchical clustering of atom-pair fingerprints highlights structural relationships among the six screened flavonoids. Bavachinin forms the most distant branch, indicating low similarity to the remaining compounds, whereas oroxylin A cluster most closely with baicalein and wogonin, consistent with their shared flavone scaffold. Heights on the y-axis represent 1 − Tanimoto similarity (average-linkage clustering) **(B)** Heatmap showing pairwise Tanimoto similarity values computed from atom-pair for the six flavonoids. Warmer colors indicate higher structural similarity, whereas lighter colors denote lower similarity. Oroxylin A and baicalein display the highest similarity and cluster together with wogonin, while bavachinin shows uniformly low similarity to the other ligands and forms a separate branch in the accompanying dendrogram.

The chemical similarity result showed bavachinin was found to be the most divergent, with the relatively close clustering of eupalitin and apigenin and a separate cluster of wogonin, baicalein, and oroxylin A. (Figure 9A).

Notably, the structural clustering in Figure 9A is not expected to mirror the ADME–toxicity PCA in Figure 8 exactly. Compounds that are structurally related (oroxylin A, baicalein, and wogonin), shown in Figure 9A can still occupy distinct regions of ADME–toxicity space as in Figure 8 due to relatively small differences in substitution patterns that alter solubility, lipophilicity, or predicted organ toxicities. Conversely, bavachinin appears as an outlier in both analyses, reflecting its distinct chemotype as well as its more extreme physicochemical and toxicity profile.

The Tanimoto similarity heatmap provided a confirmatory result for the clustering observed in the dendrogram and identified unique structural clusters within the six flavonoids under study (Figure 9B).

### 3.9 Prediction of oroxylin a targets

We used SwissTargetPrediction to predict the human targets for oroxylin A. A total of 100 human targets for oroxylin A were predicted based on its chemical structure and similarity to known ligands (Supplementary Table S4).

### 3.10 Immune-related pathways enriched among predicted targets of oroxylin A

**FIGURE 10:**
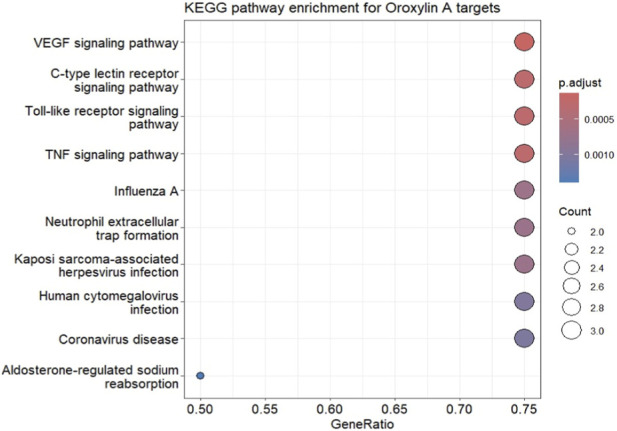
KEGG pathway enrichment of predicted human targets of oroxylin A. Dot plot showing KEGG pathways significantly enriched among the predicted human targets of oroxylin A. Each dot is positioned by the gene ratio on the x-axis, and the color reflects the adjusted p value (p. adjust). Immune- and cytokine-related pathways, including VEGF signaling, C-type lectin receptor signaling, Toll-like receptor signaling, and TNF signaling, show high fold enrichment and low adjusted p values, whereas several viral infection pathways and neutrophil extracellular trap formation are also significantly enriched.

KEGG enrichment analysis of predicted targets of oroxylin A identified significant overrepresented pathways, which were mostly related to immune signaling (Figure 10). The top overrepresented pathways were related to VEGF signaling, C-type lectin receptor signaling, Toll-like receptor signaling, and TNF signaling. These enrichment results indicate that immune-related pathways dominate the KEGG profile of predicted oroxylin A targets, suggesting that the lead chemotype is embedded in a network of signaling pathways closely linked to inflammatory responses.

### 3.11 Independent transcriptomic validation of predicted oroxylin targets in SLE blood cohorts

#### 3.11.1 Differential expression results of the SLE cohort

**FIGURE 11:**
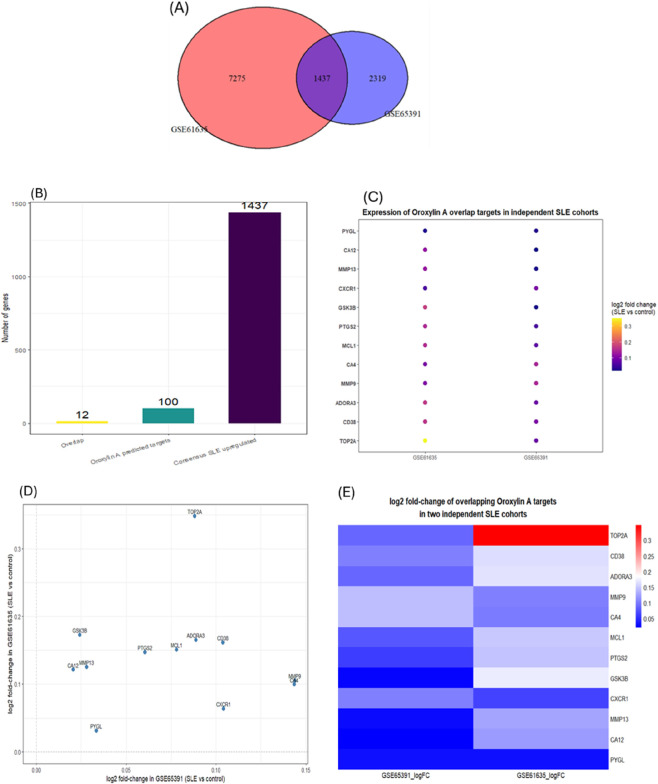
Independent transcriptomic validation and concordant expression profiling of predicted oroxylin A targets in public systemic lupus erythematosus cohorts. **(A)** Consensus SLE Upregulated Signature: A *venn diagram demonstrating the intersection of significantly upregulated genes between two independent whole-blood datasets, GSE61635 and GSE65391. The intersection defines a robust, reproducible signature of 1,437 consensus upregulated genes.* **(B)** Target Intersection Diagnostics: *Distribution bar plot illustrating the total numbers of consensus SLE-upregulated genes (1,437), computationally predicted human targets of oroxylin A (100), and their resulting overlap (12 genes). This intersection indicates that 12% of the oroxylin A target network maps directly onto reproducible clinical transcriptional changes.* **(C)** Cross-Cohort Expression Directionality: *Dot plot detailing the individual \log_2 fold changes of the 12 overlapping genes across both cohorts. All overlapping targets lie within positive values, confirming consistent, synchronized upregulation in SLE patients relative to healthy controls. The point color scale corresponds to the magnitude of upregulation.* **(D)** Quadrant Correlation Assessment: *Scatterplot comparing the calculated \log_2 fold changes between GSE65391 (x-axis) and GSE61635 (y-axis) for the 12 intersecting genes. Localization of the targets within the upper-right quadrant mathematically confirms strong structural concordance and directional alignment across the distinct observational platforms.* **(E)** Differential Expression Heatmap: Two-dimensional hierarchically structured heatmap highlighting the comparative magnitude of upregulation across datasets. *Color scale maps relative z-scores (red: maximum upregulation, blue: minimal positive fold change). Proliferation, matrix remodeling, and immune mediators like TOP2A, CD38, ADORA3, and MMP9 emerge as the most prominently overexpressed features across both patient cohorts*.

We identified 9,594 genes upregulated in SLE versus healthy controls in GSE61635 and 3,756 genes upregulated in GSE65391 (adjusted p < 0.05 and log2 fold change >0). Their intersection comprised 1,437 genes that were consistently upregulated in both cohorts, representing a consensus SLE upregulated signature (Figure 11A).

#### 3.11.2 Overlapped genes between oroxylin a targets and upregulated genes in SLE cohorts

When the 100 predicted human targets of oroxylin A (from SwissTargetPrediction) were intersected with the consensus SLE upregulated gene set (1,437 genes consistently upregulated in both GSE65391 and GSE61635), we identified 12 overlapping genes that were both predicted targets and reproducibly upregulated in SLE whole blood transcriptomics (ADORA3, CA12, CA4, CD38, CXCR1, GSK3B, MCL1, MMP13, MMP9, PTGS2, PYGL, and TOP2A) (Figure 11B). This represents a relatively small fraction of the consensus SLE signature, indicating a modest but non-random convergence between the similarity-predicted target set and SLE-associated transcriptomics.

To further characterize these overlapping genes, the log2 fold changes from GSE65391 and GSE61635 were visualized. A dot plot demonstrated that all 12 overlap genes showed positive log2 fold changes in both datasets, indicating consistent upregulation in SLE relative to healthy controls (Figure 11C). Scatterplot comparison of GSE65391 versus GSE61635 log2 fold changes showed that most overlapping genes lay in the upper-right quadrant, confirming their expression across both datasets. TOP2A, CD38 and ADORA3 exhibited the largest combined log2 fold changes, whereas metabolic and matrix-associated genes such as PYGL, MMP9 and MMP13 also showed reproducible, more modest upregulation (Figure 11D).

A heatmap summarizing log2 fold changes for all 12 overlap genes in both cohorts highlighted TOP2A, CD38, ADORA3 and MMP9 among the most strongly upregulated targets, with broadly similar patterns across both datasets (Figure 11E).

## 4 Discussion

In this *in silico* study, all six flavonoids examined (apigenin, baicalein, bavachinin, eupalitin, oroxylin A, and wogonin) were shown to form favorable docking to TLR7, indicating a broad compatibility in terms of TLR7 targeting.

Bavachinin produced the highest score in docking simulation, while oroxylin A and eupalitin also performed better than baicalein, apigenin, and wogonin. The docking revealed bavachinin was the top-ranked TLR7 ligand, showing the lowest binding energy (−9.80 kcal/mol) with three hydrogen bonds within the receptor pocket. Oroxylin A and eupalitin also performed well in docking simulation (−8.48 and −7.87 kcal/mol, respectively). While baicalein, apigenin, and wogonin were predicted to have less favorable docking energy (Figure 3) and form fewer hydrogen bonds (Table 1). This result is in agreement with previous findings on the capability of flavonoids to produce favorable docking energy via simultaneous establishment of hydrogen bonds and hydrophobic contacts in nucleic acid–binding pockets (Liu et al., 2025; Shen et al., 2022; Tang et al., 2025). However, MD simulations carried out as part of the current investigation demonstrate that good docking energy does not guarantee long-term stability of a ligand–protein complex.

Moreover, in Table 1 and Figure 1, Analysis of the interaction profiles revealed that several ligands shared common binding residues within the TLR7 binding pocket. Notably, residues such as Ile805, Lys54, and Tyr807 were recurrently involved in ligand binding, suggesting that these amino acids play important roles in stabilizing ligand–receptor complexes. The repeated occurrence of these residues among different ligands indicates that the compounds likely occupy overlapping binding regions within the receptor cavity. Furthermore, several ligand pairs, including Wogonin and Oroxylin A, Baicalein, Apigenin, and Bavachinin, as well as Eupalitin and Bavachinin, exhibited similar interaction patterns and shared key residues. This observation may be attributed to the structural similarities among these flavonoid compounds, which contain related aromatic ring systems and hydroxyl substitution patterns that favor comparable binding orientations within the receptor pocket. The conservation of these interactions suggests that the identified residues constitute critical recognition elements for flavonoid binding to TLR7. The presence of overlapping binding residues may also imply the potential for competitive binding among these compounds if administered simultaneously. However, further molecular dynamics simulations, binding free-energy calculations, and experimental validation would be required to determine whether such competition occurs under physiological conditions and whether it may influence the therapeutic efficacy of combination treatments.

Beyond static docking scores, the molecular dynamics simulations were crucial for discriminating between bavachinin and oroxylin A. While bavachinin achieved the most favorable initial docking energy, the 100-ns MD trajectories showed that the oroxylin A–TLR7 complex maintained lower backbone RMSD values and more persistent hydrogen-bond and hydrophobic interactions in the binding pocket, indicating greater conformational stability over time (Figure 4). This illustrates the added value of MD as a complementary step to docking in natural product screening. In the context of structurally related flavonoids, such an integrated docking–MD workflow therefore allows prioritization of the potential leads like oroxylin A that combine strong binding with robust dynamic stability, rather than relying solely on docking energies.

Consideration of ADME–toxicity parameters, multivariate analysis, and chemotype properties demonstrated that oroxylin A was characterized by a favorable balance of physicochemical characteristics and druglike and safe properties associated with the flavone skeleton and involvement of immune- and cytokine-related pathways.

The obtained results from ADME and toxicity studies indicate that both bavachinin and oroxylin A are characterized by relatively low toxicity and physicochemical characteristics suitable for oral administration, as both bavachinin and oroxylin A satisfy Lipinski’s rule (Supplementary Table S2). However, toxicity prediction using ProTox II demonstrates that both bavachinin and oroxylin A are characterized by organ-specific toxicity risks. Both compounds carry respiratory toxicity risks, while bavachinin is predicted to be immunotoxic and oroxylin A cardiotoxic, but neither of these two compounds is potentially toxic for the liver or cytotoxic or carcinogenic (Supplementary Table S3). The overall pattern of toxicity and physicochemical characteristics is reflected in the ADME-tox heatmap for all screened compounds, in which bavachinin stands apart with high molecular weight and logP, low TPSA, and an unusual solubility profile, likely due to its lipophilic properties and predicted immunotoxicity. Oroxylin A displays low molecular weight, moderate lipophilicity, and intermediate TPSA and hydrogen bond numbers, indicating moderate size and lipophilicity with an intermediate *in silico* toxicity profile (respiratory and cardiotoxic alerts, but not immunotoxic) (Figure 7). PCA confirms these results, showing separation of bavachinin and oroxylin A along PC1 and PC2, respectively, due to bavachinin’s large size, lipophilicity, and toxicity and oroxylin A’s intermediate lipophilicity and unique solubility pattern and organ toxicity indices. Thus, both compounds display characteristic patterns in terms of the corresponding chemotypes, with bavachinin showing more extreme characteristics and being characterized by a more lipophilic structure (Figure 8).

Chemical similarity analysis demonstrates that bavachinin forms the most distant branch within the screening dataset (Figure 9A), with low Tanimoto similarity with the other ligands (Figure 9B), reflecting structural differences in its chemotype. The remaining five compounds are grouped into a tighter cluster, with oroxylin A and baicalein/wogonin forming a smaller subcluster and apigenin/eupalitin another one. Taken together with ADME–toxicity and MD results, this clustering suggests that bavachinin’s highly lipophilic chemotype is responsible for its unique ADME profile and instability in simulations, while oroxylin A belongs to a different but more tractable chemotype, with baicalein and wogonin forming another subgroup in terms of physicochemical and biological properties.

In the ADME–toxicity analysis, oroxylin A was predicted to have a potential risk of cardiotoxicity despite an otherwise balanced physicochemical and pharmacokinetic profile. This result is in agreement with multiple studies found the cardiotoxic effect of (Lu et al., 2021)This prediction reflects structural features shared with other flavonoids that can interact with cardiac ion channels or signaling pathways *in silico*, rather than a proven *in vivo* liability (Scholz et al., 2007; Scholz et al., 2010).

Experimental data on Oroxylin A and related *Scutellaria* flavones have generally emphasized anti-inflammatory and cytoprotective effects, with limited direct evidence for clinically relevant cardiac toxicity; in particular, Oroxylin A protects against doxorubicin-induced acute cardiotoxicity in mice and exhibits low cytotoxicity while suppressing inflammatory cytokines in diverse inflammatory models (Wang et al., 2024; Zhang et al., 2021), suggesting that the *in silico* cardiotoxicity signal should be interpreted cautiously and viewed as a safety hypothesis rather than a contraindication. From a drug-development perspective, any future optimization of oroxylin A as a TLR7-directed lead would need to incorporate early cardiac safety pharmacology to confirm or reject this prediction. This makes it necessary to perform additional cardiopulmonary and immunotoxicity analysis before proceeding to *in vivo* experiments in SLE. Nevertheless, it is worth noting that *in silico* ADME and toxicity predictions are only indicative, providing probabilistic results subject to both false positives and false negatives, and thus should be used only for hypothesis generation.

KEGG enrichment analysis of the predicted human targets of oroxylin A identified several significantly enriched Immune- and cytokine-related pathways, including VEGF signaling, C-type lectin receptor signaling, Toll-like receptor signaling, and TNF signaling (Figure 10). These pathways are broadly consistent with known processes of immune activation relevant to systemic autoimmunity and SLE pathogenesis. However, this enrichment should not be interpreted as direct evidence that oroxylin A modulates TLR7 signaling or SLE-specific mechanisms *in vivo*. Instead, it is suggested that the potential targets of oroxylin A are located within signaling pathways that can modulate inflammatory and immunoregulatory pathways. Accordingly, these KEGG findings are best described as hypothesis-generating, providing candidate signaling axes for future mechanistic and experimental studies.

Additionally, we found 1,437 genes representing a consensus SLE upregulated signature that were consistently upregulated in both studies’ SLE datasets (Figure 11A). Intersecting the 100 predicted human targets of oroxylin A with the list of genes upregulated in two independent SLE GEO datasets revealed a set of 12 overlap genes (Figure 11B). These genes include mediators of proliferation such as DNA topoisomerase II (TOP2A) (Neubauer et al., 2016; Wang et al., 2022), lymphocyte activation (CD38, ADORA3) (Gessi et al., 2004; Ghosh et al., 2023; Glaría et al., 2020; Jacobson et al., 2018), matrix remodeling (MMP9, MMP13) (Lausch et al., 2009; Pag et al., 2007; Sung et al., 2005; Toriseva et al., 2007), metabolism (PYGL, CA4, CA12) (Agius, 2015; Alvarez et al., 2003; Waheed and Sly, 2017), inflammatory signaling (PTGS2, CXCR1, GSK3B) (Martín-Vázquez et al., 2023; Medunjanin et al., 2016; Toya et al., 2024), and antiapoptotic pathways (MCL1) (Carrington et al., 2017). All of which are overexpressed in SLE patients relative to healthy controls (Figures 11C,D **)**. Taken together, these results support oroxylin A as the most compelling hypothesis-generating TLR7-targeting candidate among the screened flavonoids while underscoring that all findings remain predictive and require rigorous experimental validation.

As our network analysis highlighted TOP2A as a hub associated with the TLR7-related gene signature. Consistent with this, several flavonoids such as quercetin, luteolin, myricetin, and fisetin have been experimentally shown to inhibit TOP2A activity *in vitro* (Sitarek et al., 2024). In our study, TOP2A should therefore be viewed as an unvalidated mechanistically credible target whose modulation by the flavonoids remains a testable hypothesis. CD38 emerged in our network as an immunometabolic node linked to the TLR7-centric signature. Experimental studies have demonstrated that the flavonoids apigenin and quercetin inhibit human CD38 *in vitro* and in cells (Escande et al., 2013; Kellenberger et al., 2011). Accordingly, our results should be interpreted as suggesting a shared chemotype compatible with known flavonoids. Together, the KEGG and overlap results provide indirect support that the predicted oroxylin A target network intersects immune-relevant pathways while remaining as a computational hypothesis generation for future direct experimental validation.

## 5 Conclusion

In this study, we used an integrated *in silico* pipeline to compare six related flavonoids as TLR7-binding candidates in SLE. Docking favored bavachinin, but molecular dynamics, ADME–toxicity profiling, and chemotype analysis collectively indicated that oroxylin A forms a more stable TLR7 complex, has more balanced drug-like and safety properties, and is linked to immune-related pathways. These data prioritize oroxylin A as a hypothesis-generating TLR7-targeted flavonoid lead and highlight the value of combining docking, dynamics, ADME–toxicity, chemotype, and pathway analyses for natural-product lead selection while underscoring the need for comprehensive *in vitro* and *in vivo* validation.

## 6 Limitations

This study has some limitations. First, the flavonoid set was intentionally small and focused rather than derived from a large high-throughput screening library. Accordingly, the present work should be interpreted as a targeted prioritization study designed to generate hypotheses for future experimental validation, not as a comprehensive screening. Second, all docking and molecular dynamics simulations were based on use of a single human TLR7 cryo-EM structure (PDB ID: 7CYN), which has a resolution of 4.2 Å. Although this structure provides an experimentally validated active conformation and a defined ligand-binding site, its relatively low resolution may reduce the accuracy of predicted protein–ligand interactions and docking scores. Therefore, the molecular docking results should be interpreted with caution and considered as supportive evidence that requires further validation through additional computational approaches and experimental studies. We did not perform docking using multiple TLR7 conformations or molecular dynamics snapshots to account for receptor flexibility, which limits our ability to capture induced-fit effects and alternative binding modes that may be relevant for structurally diverse flavonoids. Third, the results of the investigation are purely computational with no *in vitro* and *in vivo* experimental validation steps. Although this study studied TLR7-binding flavonoids, we did not directly assess binding selectivity against closely related endosomal receptors such as TLR8 and TLR9, which are known to interact functionally with TLR7 in autoimmune signaling; thus, receptor selectivity remains an important question for future experimental validation.

## Data Availability

The original contributions presented in the study are included in the article/Supplementary Material, further inquiries can be directed to the corresponding author.
