# Supplementary material for: Integrated docking–molecular dynamics, ADME–toxicity profiling, and transcriptomic validation identify oroxylin a as a TLR7-targeting flavonoid candidate in systemic lupus erythematosus
Source: Front Chem. 2026 Jul 1;14:1863132. doi: 10.3389/fchem.2026.1863132 (PMC13368978; doi:10.3389/fchem.2026.1863132)
Supplement: Supplementary file 1 [file Supplementaryfile1.docx]

**Table S1. PubChem Compound IDs (CIDs), KEGG Compound IDs, and corresponding web links for the investigated phytochemical ligands.**

| Compound | PubChem CID | Weblink |
| --- | --- | --- |
| Baicalein | 5281605 | <https://pubchem.ncbi.nlm.nih.gov/compound/5281605> |
| Apigenin | 5280443 | <https://pubchem.ncbi.nlm.nih.gov/compound/5280443> |
| Wogonin | 5281703 | <https://pubchem.ncbi.nlm.nih.gov/compound/5281703> |
| Oroxylin A | 5320315 | <https://pubchem.ncbi.nlm.nih.gov/compound/5320315> |
| Eupalitin | 5748611 | <https://pubchem.ncbi.nlm.nih.gov/compound/5748611> |
| Bavachinin | 10337211 | <https://pubchem.ncbi.nlm.nih.gov/compound/10337211> |
| Oroxylin A (KEGG) | C22603 | <https://www.kegg.jp/entry/C22603> |

**Table S2.** Lipinski’s Rule of Five parameters and predicted solubility profiles for Bavachinin and Oroxylin A obtained from SwissADME.

| Phytocompound | Physicochemical properties | | | | Lipophilicity | Drug likeness/  Lipinski’s rule | Solubility | | |
| --- | --- | --- | --- | --- | --- | --- | --- | --- | --- |
|  | Molecular  weight | Hydrogen  bond  donors | Hydrogen  bond acceptors | Molar  Refractivity |  |  | ESOL  class | Ali  class | Silicos-  IT class |
| Bavachinin | 338.40  g/mol | 1 | 4 | 97.74 | 3.47 | Yes; 0 violation | -4.83 | -5.34 | -5.93 |
| Oroxylin A | 284.26 g/mol | 2 | 5 | 78.46 | 2.61 | Yes; 0 violation | -4.23 | -4.85 | -5.10 |

ESOL: predicts aqueous solubility from molecular structure

**Table S3.** Toxicity profiles of Bavachinin and Oroxylin A predicted using PROTOX-II.

| compound | Predicted  LD50 (mg/kg) | Predicted  toxicity  class | Hepato-  toxicity | Neuro toxicity | Carcinogenicity | Mutagenicity | Immunotoxicity | Respiratory toxicity | Cardiotoxicity |
| --- | --- | --- | --- | --- | --- | --- | --- | --- | --- |
| Bavachinin | 2000mg/kg | 4 | _ | _ | _ | _ | + | + | _ |
| Oroxylin A | 4000mg/kg | 5 | _ | _ | _ | _ | _ | + | + |

*LD50: Lethal Dose 50%, +, Active; −, inactive.*

**Table S4. Oroxylin A targets predicted by SwissTargetPrediction.**

| Target | Common name | Uniprot ID | Probability* |
| --- | --- | --- | --- |
| Tyrosine-protein kinase receptor FLT3 | FLT3 | P36888 | 1 |
| Nitric oxide synthase, inducible (by homology) | NOS2 | P35228 | 0.6004118 |
| Cyclooxygenase-2 (by homology) | PTGS2 | P35354 | 0.270836923 |
| P-glycoprotein 1 | ABCB1 | P08183 | 0.22133019 |
| Stem cell growth factor receptor | KIT | P10721 | 0.196657136 |
| Delta opioid receptor | OPRD1 | P41143 | 0.196657136 |
| Lysine-specific demethylase 4D-like | KDM4E | B2RXH2 | 0.180252494 |
| Xanthine dehydrogenase | XDH | P47989 | 0.180252494 |
| Arachidonate 15-lipoxygenase | ALOX15 | P16050 | 0.180252494 |
| Cyclin-dependent kinase 1 | CDK1 | P06493 | 0.180252494 |
| Arachidonate 12-lipoxygenase | ALOX12 | P18054 | 0.180252494 |
| G protein-coupled receptor kinase 6 | GRK6 | P43250 | 0.180252494 |
| Serine/threonine-protein kinase PIM1 | PIM1 | P11309 | 0.171978589 |
| Adenosine A1 receptor (by homology) | ADORA1 | P30542 | 0.171978589 |
| Adenosine A2a receptor (by homology) | ADORA2A | P29274 | 0.171978589 |
| Estrogen receptor beta | ESR2 | Q92731 | 0.139061947 |
| Aldose reductase (by homology) | AKR1B1 | P15121 | 0.139061947 |
| Tyrosine-protein kinase LCK | LCK | P06239 | 0.130791955 |
| Receptor-type tyrosine-protein phosphatase S | PTPRS | Q13332 | 0.122581769 |
| Estrogen receptor alpha | ESR1 | P03372 | 0.122581769 |
| Adenosine A3 receptor | ADORA3 | P0DMS8 | 0.122581769 |
| Cytochrome P450 1B1 | CYP1B1 | Q16678 | 0.114337559 |
| Estradiol 17-beta-dehydrogenase 1 | HSD17B1 | P14061 | 0.114337559 |
| Monoamine oxidase A | MAOA | P21397 | 0.114337559 |
| Tyrosine-protein kinase SYK | SYK | P43405 | 0.114337559 |
| Aldo-keto reductase family 1 member B10 | AKR1B10 | O60218 | 0.114337559 |
| ATP-binding cassette sub-family G member 2 | ABCG2 | Q9UNQ0 | 0.114337559 |
| Androgen Receptor | AR | P10275 | 0.114337559 |
| Plasminogen | PLG | P00747 | 0.106099949 |
| Cyclin-dependent kinase 5/CDK5 activator 1 | CDK5R1 CDK5 | Q15078 Q00535 | 0.106099949 |
| Cytochrome P450 19A1 | CYP19A1 | P11511 | 0.106099949 |
| Carbonic anhydrase II | CA2 | P00918 | 0.106099949 |
| Cyclin-dependent kinase 1/cyclin B | CCNB3 CDK1 CCNB1 CCNB2 | Q8WWL7 P06493 P14635 O95067 | 0.106099949 |
| Carbonic anhydrase VII | CA7 | P43166 | 0.106099949 |
| Cyclin-dependent kinase 6 | CDK6 | Q00534 | 0.106099949 |
| Carbonic anhydrase I | CA1 | P00915 | 0.106099949 |
| Carbonic anhydrase XII | CA12 | O43570 | 0.106099949 |
| Carbonic anhydrase IX | CA9 | Q16790 | 0.106099949 |
| Carbonic anhydrase IV | CA4 | P22748 | 0.106099949 |
| Carbonyl reductase [NADPH] 1 | CBR1 | P16152 | 0.106099949 |
| Inhibitor of nuclear factor kappa B kinase beta subunit | IKBKB | O14920 | 0.106099949 |
| Neurotrophic tyrosine kinase receptor type 2 | NTRK2 | Q16620 | 0.106099949 |
| Ornithine decarboxylase | ODC1 | P11926 | 0.106099949 |
| Matrix metalloproteinase 12 | MMP12 | P39900 | 0.106099949 |
| Lymphocyte differentiation antigen CD38 | CD38 | P28907 | 0.106099949 |
| DNA topoisomerase I (by homology) | TOP1 | P11387 | 0.106099949 |
| Arginase-1 (by homology) | ARG1 | P05089 | 0.106099949 |
| Matrix metalloproteinase 9 | MMP9 | P14780 | 0.106099949 |
| Matrix metalloproteinase 2 | MMP2 | P08253 | 0.106099949 |
| PI3-kinase p110-gamma subunit | PIK3CG | P48736 | 0.097874534 |
| Glycogen synthase kinase-3 beta | GSK3B | P49841 | 0.097874534 |
| Multidrug resistance-associated protein 1 | ABCC1 | P33527 | 0.097874534 |
| Calmodulin | CALM1 | P62158 | 0.097874534 |
| Phospholipase A2 group IIA | PLA2G2A | P14555 | 0.097874534 |
| Acetylcholinesterase | ACHE | P22303 | 0.097874534 |
| Solute carrier family 22 member 12 | SLC22A12 | Q96S37 | 0.097874534 |
| Telomerase reverse transcriptase | TERT | O14746 | 0.097874534 |
| AMY1C | AMY1A | P04745 | 0.097874534 |
| Tankyrase-1 | TNKS | O95271 | 0.097874534 |
| Transthyretin | TTR | P02766 | 0.097874534 |
| Estradiol 17-beta-dehydrogenase 2 | HSD17B2 | P37059 | 0.097874534 |
| Tyrosinase | TYR | P14679 | 0.097874534 |
| Aryl hydrocarbon receptor | AHR | P35869 | 0.097874534 |
| Estrogen-related receptor alpha | ESRRA | P11474 | 0.097874534 |
| Phosphodiesterase 5A | PDE5A | O76074 | 0.097874534 |
| Beta amyloid A4 protein | APP | P05067 | 0.097874534 |
| Induced myeloid leukemia cell differentiation protein Mcl-1 | MCL1 | Q07820 | 0.097874534 |
| NEDD8-activating enzyme E1 regulatory subunit | NAE1 | Q13564 | 0.097874534 |
| Epidermal growth factor receptor erbB1 | EGFR | P00533 | 0.097874534 |
| Sigma opioid receptor | SIGMAR1 | Q99720 | 0.097874534 |
| Tankyrase-2 | TNKS2 | Q9H2K2 | 0.097874534 |
| NADPH oxidase 4 | NOX4 | Q9NPH5 | 0.097874534 |
| Cytochrome P450 1A1 | CYP1A1 | P04798 | 0.097874534 |
| Mu opioid receptor | OPRM1 | P35372 | 0.097874534 |
| Interleukin-8 receptor A | CXCR1 | P25024 | 0.097874534 |
| Casein kinase II alpha | CSNK2A1 | P68400 | 0.097874534 |
| Microtubule-associated protein tau | MAPT | P10636 | 0.097874534 |
| DNA topoisomerase II alpha | TOP2A | P11388 | 0.097874534 |
| Insulin receptor | INSR | P06213 | 0.097874534 |
| Myosin light chain kinase, smooth muscle | MYLK | Q15746 | 0.097874534 |
| Myeloperoxidase | MPO | P05164 | 0.097874534 |
| PI3-kinase p85-alpha subunit | PIK3R1 | P27986 | 0.097874534 |
| Death-associated protein kinase 1 | DAPK1 | P53355 | 0.097874534 |
| Liver glycogen phosphorylase | PYGL | P06737 | 0.097874534 |
| Matrix metalloproteinase 13 | MMP13 | P45452 | 0.097874534 |
| Matrix metalloproteinase 3 | MMP3 | P08254 | 0.097874534 |
| Carbonic anhydrase III | CA3 | P07451 | 0.097874534 |
| Carbonic anhydrase XIV | CA14 | Q9ULX7 | 0.097874534 |
| Carbonic anhydrase XIII (by homology) | CA13 | Q8N1Q1 | 0.097874534 |
| Phospholipase A2 group 1B | PLA2G1B | P04054 | 0.097874534 |
| Carbonic anhydrase VA | CA5A | P35218 | 0.097874534 |
| DNA-(apurinic or apyrimidinic site) lyase | APEX1 | P27695 | 0.097874534 |
| Aldo-keto reductase family 1 member C2 (by homology) | AKR1C2 | P52895 | 0.097874534 |
| Aldo-keto reductase family 1 member C1 (by homology) | AKR1C1 | Q04828 | 0.097874534 |
| Aldo-keto-reductase family 1 member C3 (by homology) | AKR1C3 | P42330 | 0.097874534 |
| Aldo-keto reductase family 1 member C4 (by homology) | AKR1C4 | P17516 | 0.097874534 |
| Aldehyde reductase (by homology) | AKR1A1 | P14550 | 0.097874534 |
| DNA-3-methyladenine glycosylase | MPG | P29372 | 0.097874534 |
| G-protein coupled receptor 35 | GPR35 | Q9HC97 | 0.097874534 |
| Butyrylcholinesterase | BCHE | P06276 | 0.097874534 |

**
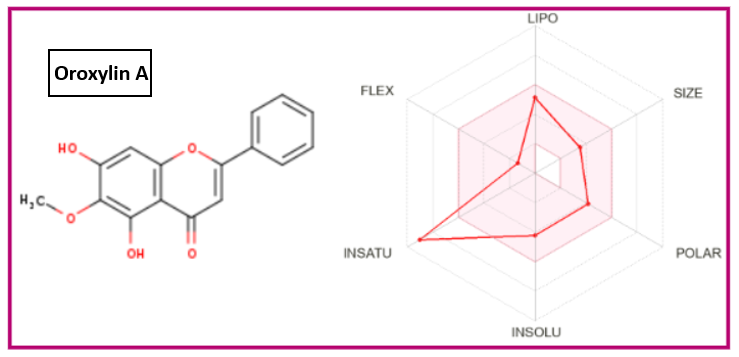
**

**Figure S1.** Physicochemical radar chart of Oroxylin A.


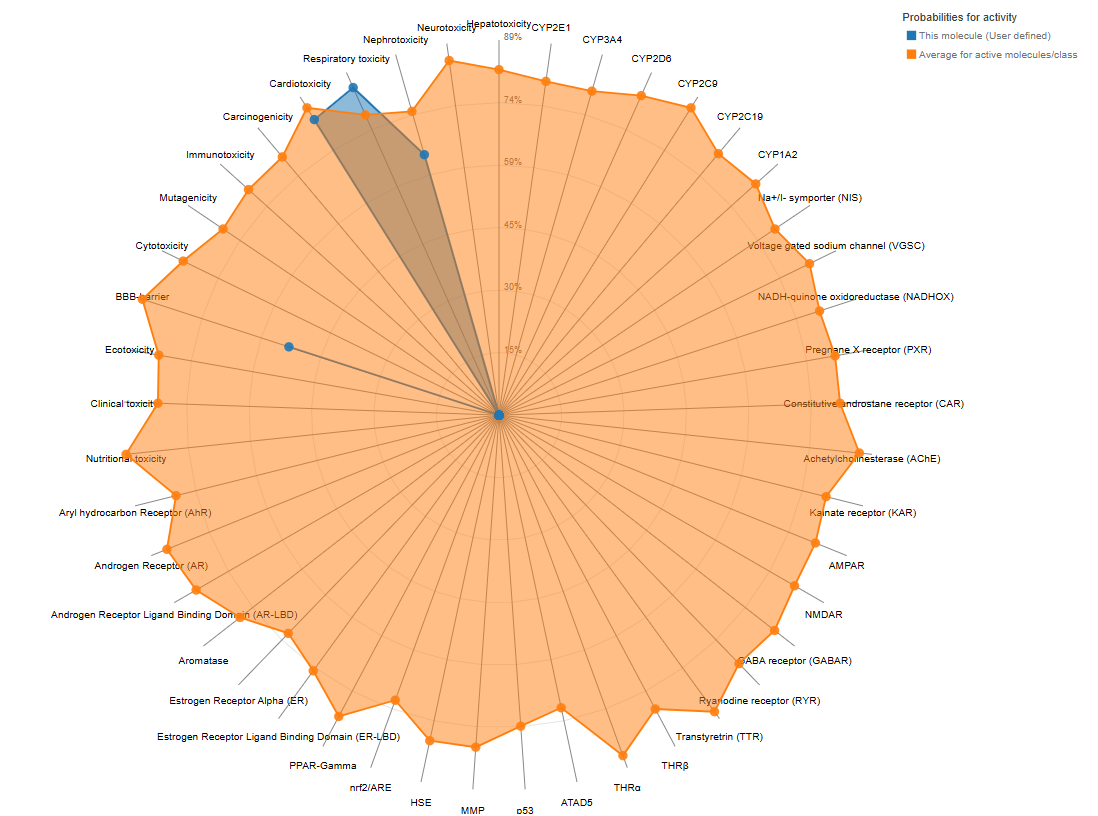


**Figure S2**. A radar chart compares the predicted toxicities of a specific molecule, Oroxylin A, against the average for a class of active molecules.
